# Visual perceptual training reconfigures post-task resting-state functional connectivity with a feature-representation region

**DOI:** 10.1371/journal.pone.0196866

**Published:** 2018-05-09

**Authors:** Mitra Taghizadeh Sarabi, Ryuta Aoki, Kaho Tsumura, Ruedeerat Keerativittayayut, Koji Jimura, Kiyoshi Nakahara

**Affiliations:** 1 School of Information, Kochi University of Technology, Kami-city, Kochi, Japan; 2 Research Center for Brain Communication, Kochi University of Technology, Kami-city, Kochi, Japan; 3 Department of Biosciences and Informatics, Keio University, Yokohama-city, Kanagawa, Japan; University of Pennsylvania, UNITED STATES

## Abstract

The neural mechanisms underlying visual perceptual learning (VPL) have typically been studied by examining changes in task-related brain activation after training. However, the relationship between post-task “offline” processes and VPL remains unclear. The present study examined this question by obtaining resting-state functional magnetic resonance imaging (fMRI) scans of human brains before and after a task-fMRI session involving visual perceptual training. During the task-fMRI session, participants performed a motion coherence discrimination task in which they judged the direction of moving dots with a coherence level that varied between trials (20, 40, and 80%). We found that stimulus-induced activation increased with motion coherence in the middle temporal cortex (MT+), a feature-specific region representing visual motion. On the other hand, stimulus-induced activation decreased with motion coherence in the dorsal anterior cingulate cortex (dACC) and bilateral insula, regions involved in decision making under perceptual ambiguity. Moreover, by comparing pre-task and post-task rest periods, we revealed that resting-state functional connectivity (rs-FC) with the MT+ was significantly increased after training in widespread cortical regions including the bilateral sensorimotor and temporal cortices. In contrast, rs-FC with the MT+ was significantly decreased in subcortical regions including the thalamus and putamen. Importantly, the training-induced change in rs-FC was observed only with the MT+, but not with the dACC or insula. Thus, our findings suggest that perceptual training induces plastic changes in offline functional connectivity specifically in brain regions representing the trained visual feature, emphasising the distinct roles of feature-representation regions and decision-related regions in VPL.

## Introduction

Repeated exposure to a specific visual feature improves perceptual sensitivity and behavioural accuracy to the trained feature [1–3]. This process is known as visual perceptual learning (VPL), and is considered an effective tool for exploring experience-dependent plasticity in the brain [4–6]. Previous studies using functional magnetic resonance imaging (fMRI) in humans have revealed that different brain regions contribute to VPL in distinct ways, depending on their specialized roles [7,8]. These studies have typically investigated changes in brain responses to specific visual stimuli over the course of perceptual learning. For instance, several studies have shown that stimulus-induced activation in brain regions representing specific visual features (e.g., the early visual cortex) tends to increase after intensive perceptual training [2,9], whereas activation in regions related to higher-order cognitive processes tends to decrease after training [9]. Other studies have reported refinements in neural representations of trained visual features after VPL, indicating training-induced plasticity in feature-representation regions [5,8]. While these findings have provided useful insights into the “online” processes supporting VPL, it is also known that “offline” processes after task completion (e.g., consolidation during sleep) play critical roles [10,11]. However, because the majority of existing fMRI studies have exclusively investigated brain activation during task periods, the contribution of offline mechanisms to VPL remains to be elucidated.

The importance of post-task offline processes in VPL has typically been studied by focusing on sleep-related consolidation processes [11,12]. However, recent studies have also highlighted the importance of wakeful resting periods immediately after training [13–15]. Neuroimaging studies utilising resting-state functional connectivity (rs-FC) [16–18] are particularly useful for investigating experience-dependent reorganisation in the brain after performing cognitive tasks. Evidence from diverse domains of cognitive neuroscience research has suggested that post-task rs-FC changes reflect recent visual/cognitive experiences [14,19], and further predict subsequent performance of memory and learning [15,20]. Studies of episodic memory are notable examples, showing that rs-FC between category-selective regions (e.g., the fusiform face area) and memory-related regions (e.g., the hippocampus) during wakeful rest immediately after performing encoding tasks is predictive of subsequent memory performance [13,21]. Although VPL and episodic memory formation are supported by different neural substrates, both involve experience-induced plasticity as underlying mechanisms [10,22,23]. This raises the possibility that rs-FC during wakeful rest immediately after training may also play a key role in VPL.

Only a few studies have investigated training-induced rs-FC changes after VPL, and the results have been mixed. One study examined rs-FC before and after intensive training on a visual shape discrimination task, finding that rs-FC between the visual feature representation region (i.e., V3) and the dorsal attention system (e.g., the frontal eye field and superior parietal lobule) decreased after training [24]. However, this study investigated the effects of intensive training over several days (2–9 days), and did not examine rs-FC changes in the early learning phase. In contrast, another fMRI study used visual motion discrimination training with a much shorter timescale (~90 min), finding that rs-FC between the hippocampus and striatum increased during a wakeful rest period immediately after training [25]. While this study revealed rapid reorganisation of rs-FC after a brief period of perceptual training, no rs-FC changes were detected in the visual feature representation region, unlike the earlier study. Thus, it remains unclear whether visual feature representation regions show training-induced rs-FC changes immediately after training.

**Fig 1:**
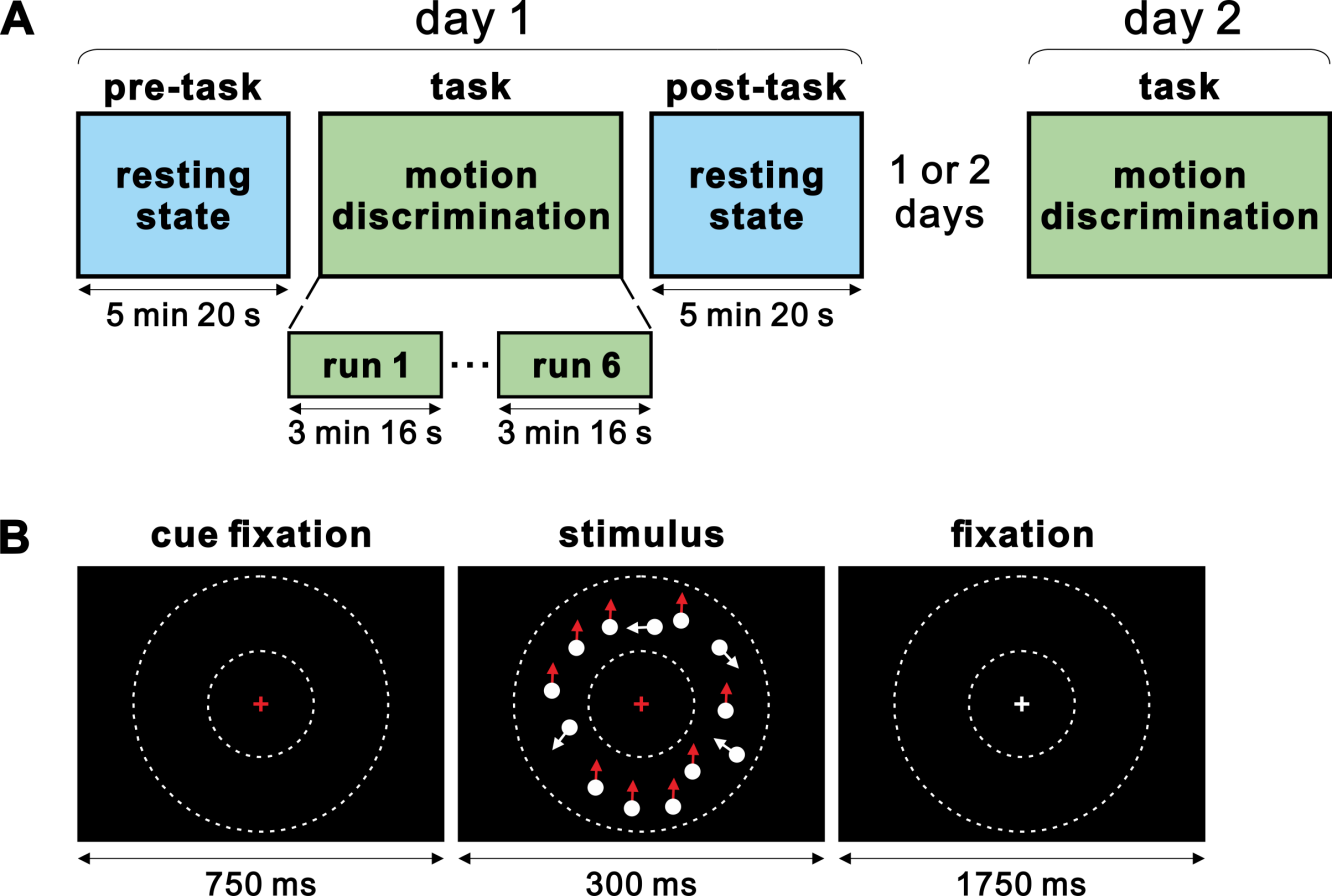
Experimental procedure and single trial of motion discrimination task. (A) Experiments were conducted over 2 days: day 1 and day 2. On day 1, fMRI scanning started with pre-task resting-state scans, followed by motion discrimination task scans, and post-task resting-state scans. On day 2, the same motion discrimination task was administered in the scanner. In each t-fMRI session (day 1 and 2), participants performed six runs of the motion discrimination task. (B) Each trial of the motion discrimination task began with 750-ms presentation of a red fixation cross cueing a subsequent visual stimulus comprised of coherently moving dots for 300 ms. The coherence level (20, 40, or 80%) and motion direction (upward or downward) varied from trial to trial. The color of the fixation cross became white when the dot stimuli disappeared and remained white for 1750 ms. Participants were required to press the corresponding button with their right thumb as quickly and accurately as possible within a response window of 1050 ms. The area in which moving dots were presented is schematically indicated in between the small and large dotted circles; these circles were not presented in actual experiments.

In the current fMRI study (Fig 1), we examined whether a brief period of visual perceptual training induced rapid reorganisation of rs-FC changes immediately after training in the visual feature representation regions. Participants were trained on a visual motion discrimination task for a short period (~30 min), in which they judged the direction of coherently moving dots randomly chosen from three coherence levels (20, 40 and 80%). We localized the MT+, a brain region representing visual motion [26–33], by analysing the parametric effects of motion coherence on stimulus-induced activation during the task fMRI (t-fMRI) session. Importantly, we obtained resting-state fMRI (rs-fMRI) scans before and immediately after the task to examine whether a brief period of visual motion discrimination training induces rs-FC changes with the MT+. Furthermore, we examined whether training-induced rs-FC changes immediately after training were associated with subsequent performance improvement. This question was inspired by recent reports that experience-induced rs-FC changes shortly after memory encoding tasks predict subsequent memory performance in later periods (~24 hours) [21]. To examine this issue, we invited the same participants back on the second day of the experiment to perform the motion discrimination task in the scanner.

## Materials and methods

### Participants

Twenty-one healthy right-handed participants with normal or corrected-to-normal vision were recruited for the experiment. All participants were native Japanese speakers with no history of neuropsychiatric disorders or current use of psychoactive medications. This study was approved by the Research Ethics Review Committee of Kochi University of Technology. All participants provided written informed consent according to guidelines approved by the Research Ethics Review Committee of Kochi University of Technology. Participants received 1000 yen per hour for participation. One participant was excluded due to being scanned on day 2 after 30 days from day 1 experiment (see experimental protocol), while all other participants were scanned on day 2 after 1 or 2 days apart (Fig 1A). Thus, the data from 20 participants (mean age 18.6 years, range 18–21; eight females) were analysed and reported in the study.

### Experimental procedure

The experiment was conducted in four stages over 2 days (day 1 and 2). On day 1, participants were scanned during three consecutive stages: started with pre-task resting-state functional magnetic resonance imaging (rs-fMRI) followed by task-fMRI (t-fMRI) and completed with post-task rs-fMRI. A short task-practice run (without scanning) was introduced between the pre-task rs-fMRI and t-fMRI sessions. On day 2, the same participants underwent the second t-fMRI session with the same task settings used for day 1.

### Task design

In each t-fMRI session, participants performed six runs of a visual motion discrimination task, in which they judged the direction of random-dot motion (Fig 1). Randomly moving dots were presented with three coherence levels (20, 40 and 80%) and with two directions (upward and downward). Participants discriminated which of the two directions the majority of dots were moving in, by pressing one of two target buttons. Assignment of the target buttons to the motion directions was counterbalanced across participants (left and right buttons were assigned to upward and downward motion respectively, and vice versa). The target button assignment for each participant was constant for two separate t-fMRI sessions. The presentation order of the stimuli (three coherence levels and two directions) was pseudorandomised. Each run consisted of 70 trials. The first and last five trials in each run were presented with the highest coherence level (80%), and were excluded from data analysis. Thus, the middle 60 trials (20 trials for each coherence level) were included in our analysis. Participants performed a total of 360 (6 × 60) effective trials on each t-fMRI session. Each trial began with a 750-ms presentation of a red fixation cross cueing the subsequent stimulus comprised of coherently moving dots for 300 ms. The color of the fixation cross became white when the dot stimuli disappeared, and remained white for 1750 ms (Fig 1B). Participants were required to press the corresponding button with their right thumb as quickly and accurately as possible within a response window of 1050 ms (Fig 1B). The onset of the response window was matched to the timing of the dot stimuli presentation. On day 1, participants underwent a short task-practice run (46 trials; in the scanner, but without scanning) immediately before the t-fMRI session. In the task-practice run, participants performed 36 trials with a coherence level of 20, 40, or 80% between the first and last five consecutive trials of the highest coherence level (i.e., 80%). No other task practice was conducted. In rs-fMRI sessions, all participants were scanned for 5 minutes and 20 seconds before and after t-fMRI on day 1. The following instructions were given to the participants: please rest with your eyes open, and remain calm.

### Stimuli

All stimuli were generated in MATLAB version 2012a, using the Psychophysics Toolbox extension, version 3.0.10 [34,35]. The stimuli were similar to those used in a previous study of perceptual decision-making [8,36] Each motion stimulus was composed of 150 white dots moving inside a donut-shaped display patch with a white cross in the center of the patch, on a black background (Fig 1B). The display patch and cross were centered on the screen and extended from 6 to 12° of visual angle. Within the display patch, every dot moved at a speed of 10° of visual angle per second. Some dots moved coherently toward one direction, while others moved randomly. The percentage of coherently moving dots determined the coherence, which was presented with three levels (20, 40, and 80%). Dot presentation was controlled to remove local motion signals following a standard method for generating motion stimuli [27,28,37,38]. Specifically, upon stimulus onset, the dots were presented at new random locations on each of first three frames. They were relocated after two subsequent frames, so that the dots in frame 1 were repositioned in frame 4, and the dots in frame 2 were repositioned in frame 5, and so on. When repositioned, each dot was either randomly presented at the new location or aligned with the pre-determined motion direction (upward or downward), depending on the pre-determined motion strength on that trial. Each stimulus was composed of 18 video frames with 60 Hz refresh rates (i.e., 300-ms presentation).

### fMRI scanning

Participants were scanned with a 3T Siemens Verio MRI scanner with a 32-channel head coil to obtain anatomical and functional scans. High-resolution anatomical images were acquired from each participant on day 1 after the second resting scan with a magnetisation-prepared rapid acquisition gradient echo (MPRAGE) T1-weighted sequence (repetition time (TR) 2500 ms; echo time (TE) 4.32 ms; flip angel (FA) 8°; 192 slices; slice thickness 1 mm, in-plane resolution 0.9 × 0.9 mm^2^). Functional images were acquired using a multi-band acceleration echo-planar imaging sequence (TR 0.8 sec; TE 30 ms; FA 45°; 80 slices in interleaved order; slice thickness 2 mm; in-plane resolution 3 × 3 mm^2^; multiband factor 8). Each functional run during the motion discrimination task involved 245 volumes, and each resting-state acquisition involved 400 volumes. The first 10 volumes of all functional runs (task and rest) were discarded to allow for T1 equilibration of longitudinal magnetisation.

### fMRI preprocessing

Functional data were analysed using SPM12 (http://fil.ion.ucl.ac.uk/spm/software/spm12). Preprocessing of functional images (both resting scans and task scans) involved sequential realignment across volumes and runs, coregistration of functional images to anatomical images, spatial normalisation to a standard MNI template with normalisation parameters estimated for the anatomical scans, spatial smoothing with a 8-mm full-width at half-maximum (FWHM) Gaussian kernel, and resampling into 1-mm isotropic voxels. No global signal regression was performed. Because of the short TR (0.8 sec), slice time correction was not applied to the data.

### First-level analysis

A general linear model (GLM) [39] approach was used to estimate parameter values for task events. In t-fMRI analysis, the effect of interest was the influence of changing coherence levels of visual motion from trial to trial. The trials with upward motion and those with downward motion were modelled with separate regressors, each of which was modulated by the parametric effect of mean-centred coherence levels across trials. Trials in which participants did not make a button press were separately coded in the GLM as nuisance effects. Those task events were time-locked to the stimulus onset of each trial, then convolved with canonical hemodynamic response function (HRF) implemented in SPM. Additionally, six motion parameters (three translation and three rotation parameters per frame) as well as white matter (WM) and cerebrospinal fluid (CSF) signal time series were also included in GLM as nuisance effects. Parameters were then estimated for each voxel across the whole brain. In rs-fMRI analysis, time series were extracted from 5-mm radius spheres centred on individual seed coordinates (the MT+, dACC, bilateral insula, and the primary visual cortex) after regressing out the eight nuisance variables (i.e., six motion parameters, WM and CSF signal time series). The extracted seed time series was then band-pass filtered between 0.01 Hz to 0.1 Hz to reduce the effects of low frequency drift and high frequency noise [13,25,40]. Finally, a separate GLM was estimated for each seed, which included the seed time series as the regressor of interest in addition to the eight nuisance variables.

### Second-level analysis

Maps of parameter estimates were first contrasted within individual participants. The contrast maps were collected from all participants, and subjected to a group-mean one-sample t-test based on permutation methods (5000 permutations) implemented in *randomise* in the FSL suite (http://fmrib.ox.ac.uk/fsl/). Statistical significance was set at *P* < 0.05, cluster-level family-wise error (FWE) corrected (with a cluster-forming threshold of *P* < 0.001, uncorrected) across the whole brain. The peaks of significant clusters were then identified and listed. To compare task-related fMRI activation between day 1 and day 2 in specific brain regions, the mean parameter estimates (i.e., the parametric effect of the coherence level) were extracted from 5-mm radius spheres centred on individual peak coordinates separately for day 1 and day 2, then compared across participants using paired *t* tests.

### Brain-behaviour correlation analysis

To compute training-induced rs-FC changes between specific pairs of regions, we followed the method described by Tambini *et al*. (2010) [13]. First, fMRI time series were extracted from a 5-mm radius sphere centred on the peak coordinates of each of the regions identified by the rs-FC analysis with the MT+ seed (Post-task vs. Pre-task rest), after regressing out the eight nuisance variables (six motion parameters and WM/CSF signal time series). Next, Pearson correlation coefficients between the two time series (the MT+ and each region of interest) were calculated, and subsequently Fisher Z transformed. The difference in Z (Post-task minus Pre-task) was used as an individual participant’s measure of training-induced rs-FC change. Finally, Pearson correlation coefficients between the training-induced rs-FC change and accuracy change were computed across participants. Distributions of variables (behavioral and rs-FC changes) were not significantly different from normal distribution (Lilliefors test, *P* > 0.062). In additional analyses, we also used combinations of positive and negative changes of rs-FC as explanatory variables, and reaction time or the inverse efficiency (an index combining reaction time and accuracy) [25] as dependent variables.

## Results

### Behavioural results

To assess behavioural improvement after visual perceptual training, participants’ discrimination accuracy and reaction times (RTs) for coherently moving dots were compared between the 2 days of t-fMRI sessions (day 1 and 2). Participants showed a marginally significant increase in overall discrimination accuracy (day 1: 83.8 ± 8.0%, mean ± standard deviation; day 2: 86.8 ± 5.8%; paired *t* test, *t*(19) = 1.95, *P* = 0.066; Cohen’s *d*_*av*_ = 0.43) [41] and a significant decrease in overall discrimination RTs (day 1: 696 ± 70 ms; day 2: 665 ± 72 ms; paired *t* test, *t*(19) = 3.17, *P* = 0.005; Cohen’s *d*_*av*_ = 0.43). These results confirmed that a single session of brief visual perceptual training (~30 min) induced behavioural improvement that lasted over 24 hours. We also assessed whether the participants exhibited behavioural changes in terms of accuracy, RTs or inverse efficiency (IE: a combinatorial index of accuracy and RT) [25] in the course of the 6 runs of t-fMRI scan on day 1. An analysis of variance (ANOVA) showed that those behavioural indices did not show significant effects (accuracy: *F*(5,114) = 0.29, *P* = 0.91; RT: *F*(5,114) = 0.56, *P* = 0.73, IE: *F*(5,114) = 0.21, *P* = 0.96). Comparisons of the behavioural measures were also conducted specifically between the first (run 1) and the last (run 6) runs. Again, the participants showed no significant behavioural change between run 1 and 6 (accuracy: *P* = 0.268; RT: *P* = 0.29; IE: *P* = 0.76; paired *t* test).

### Task-related fMRI activation

**Fig 2:**
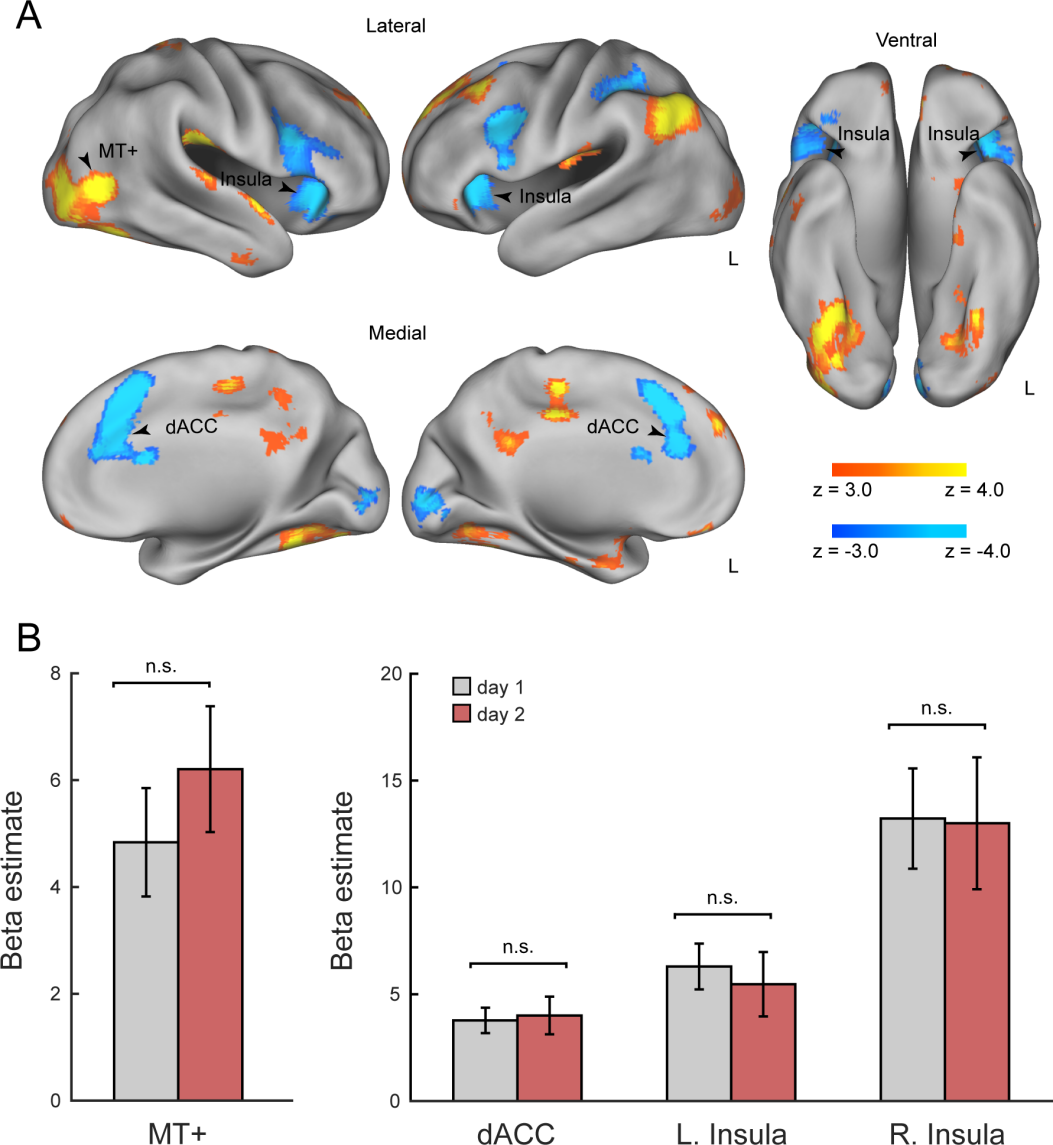
Task-related fMRI activation. (A) Positive and negative parametric effects of motion coherence. The statistical maps indicate brain regions showing significant activation increases with the coherence level (hot, including the MT+) and decreases with the coherence level (cold, including the dACC and bilateral insula). All maps are thresholded at *P* < 0.05, cluster-level FWE corrected across the whole brain. (B) Mean beta values for regions MT+, dACC, left and right insula. Peak coordinates derived from combining day 1 and day 2 t-fMRI data (Table 1). dACC: dorsal anterior cingulate cortex.

To identify brain regions involved in visual motion discrimination, we examined stimulus-induced fMRI activation that was parametrically modulated by the coherence level of moving dots. First, we combined t-fMRI data from both sessions (day 1 and 2) to probe stimulus-induced activation. As predicted, we found that stimulus-induced activation was positively modulated by the coherence level in the MT+, a region that is well established as the feature-representation region for visual motion (*P* < 0.05, cluster-level FWE corrected; see Fig 2A and Table 1). The peak MNI coordinates of the MT+ (*x* = 42, *y* = −64, *z* = 6) were close to those reported in previous studies [8,42,43]. On the other hand, stimulus-induced activation was negatively modulated by the coherence level in the dACC and bilateral insula (Fig 2A), the regions known to show task-difficulty dependent activation during perceptual decision making tasks [44,45].

**Table 1:** Regions showing significant activation by parametric effect of motion coherence during performance of direction discrimination task (cluster-level FWE-corrected *P* < 0.05).

				MNI coordinates			
Region		Hemi	#voxels	x	y	z	Peak *t*
**Positive parametric effect**							
Frontal pole		R	2758	16	50	40	7.32
Temporal occipital fusiform cortex		R	2379	26	−52	−18	7.72
	Lateral occipital cortex (MT+)	R		42	−64	6	7.59
Lateral occipital cortex		L	1525	−44	−64	42	8.89
Parietal operculum cortex		R	797	46	−24	22	7.87
Precuneus cortex		R	765	0	−54	32	5.78
Cingulate gyrus		L	668	−4	−28	42	7.48
Putamen*		L	575	−30	−16	2	6.07
Temporal occipital fusiform cortex		L	453	−30	−58	−14	5.68
Superior parietal lobule		R	334	18	−46	70	5.25
Parietal operculum cortex		L	329	−40	−28	20	5.64
Middle temporal gyrus		R	270	58	−8	−28	5.35
Lateral occipital cortex		L	248	−38	−84	−10	4.45
Frontal medial cortex		L	212	−2	46	−18	5.24
Insular cortex/putamen*		R	208	32	−16	4	5.21
Superior temporal gyrus		R	193	58	0	−4	5.42
**Negative parametric effect**							
Paracingulate gyrus (dACC)		R	2559	16	22	32	7.83
Insular cortex		R	1945	36	22	0	7.41
Insular cortex		L	1748	−26	20	2	7.41
Supramarginal gyrus		L	617	−32	−38	34	5.25
Occipital pole		R	533	−6	−92	−2	6.09

Anatomical labels derived from Harvard-Oxford cortical structural atlas.

*Labels derived from Harvard-Oxford Subcortical structural atlas. L = left hemisphere, R = right hemisphere, #voxels = number of voxels. The MT+ was identified as the second peak of the cluster.

Next, we examined whether these regions showed learning-dependent activation changes between day 1 and day 2. For this analysis, we extracted the parametric effects of coherence level from these regions separately for day 1 and day 2 (Fig 2B). No region showed a significant difference in the parametric effect of the coherence level between day 1 and day 2 (*t*(19) < 0.90, *P* > 0.382). Note that we used orthogonal contrasts for localizations of the regions (day 1 and 2, combined) and comparisons of activation (day 1 vs. day 2), thereby avoiding circular analysis. Learning-dependent activation changes also assessed in the course of t-fMRI runs on day 1. ANOVA indicated that no region exhibited significant effects across runs (MT+: *F*(5,114) = 1, *P* = 0.38; left insula: *F*(5,114) = 1.52, *P* = 0.19, right insula: *F*(5,114) = 1.27, *P* = 0.28; dACC: *F*(5,114) = 0.65, *P* = 0.66). Specific comparisons between run 1 and 6 also indicated no significant difference (MT+: *t*(19) = 0.64, *P* = 0.52; left insula: *t*(19) = −0.17, *P* = 0.86; right insula: *t*(19) = −1.05, *P* = 0.30; dACC: *t*(19) = 0.17, *P* = 0.86; paired *t* test).

### Resting-state functional connectivity

**Fig 3:**
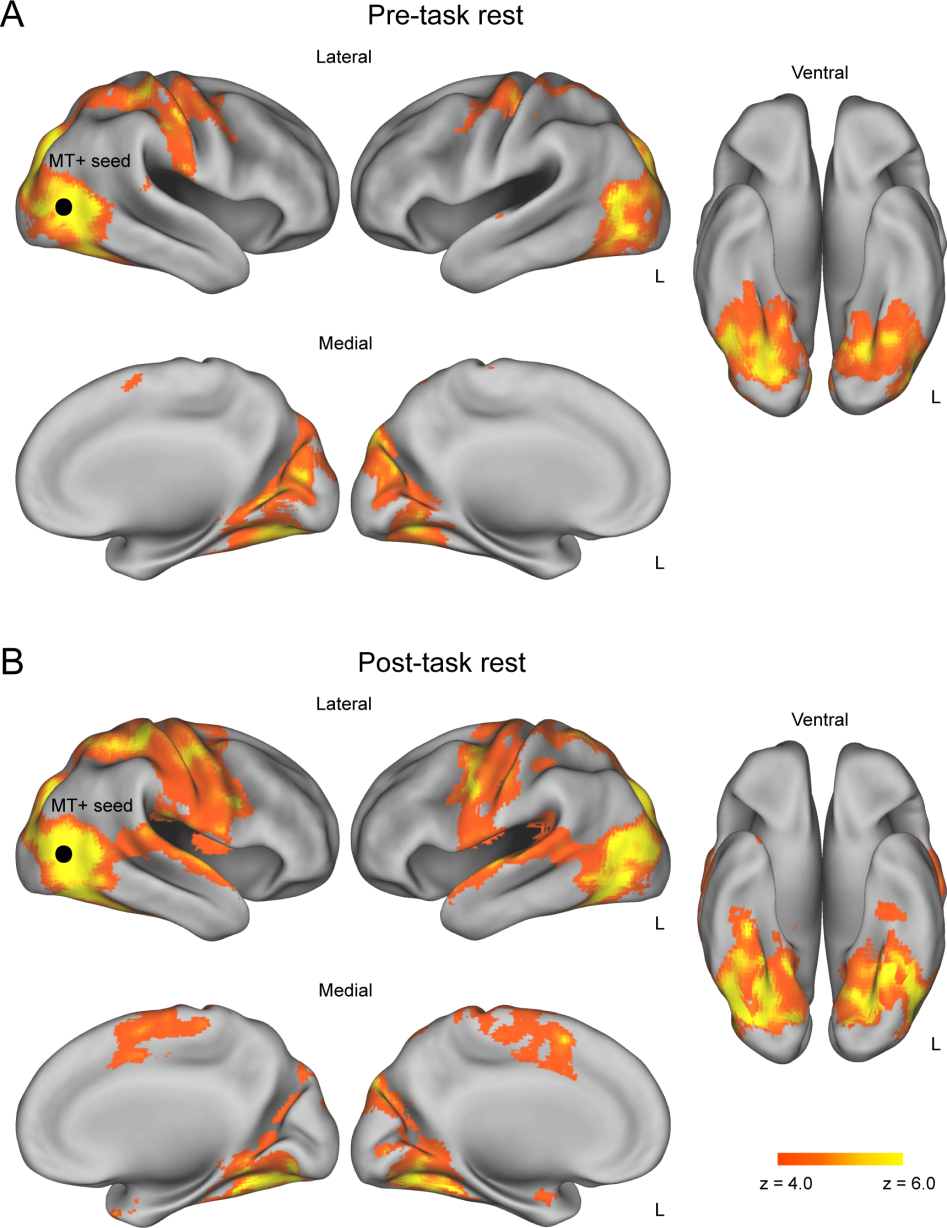
Resting-state functional connectivity with MT+ seed. (A) Pre-task rs-FC maps indicating areas that showed significant rs-FC with the MT+ before participants performed the motion discrimination task. (B) Post-task rs-FC maps indicating areas that showed significant rs-FC with the MT+ after participants performed the motion discrimination task. All maps are thresholded at *P* < 0.05, cluster-level FWE corrected across the whole brain. Black circle: approximate location of the MT+ seed.

**Fig 4:**
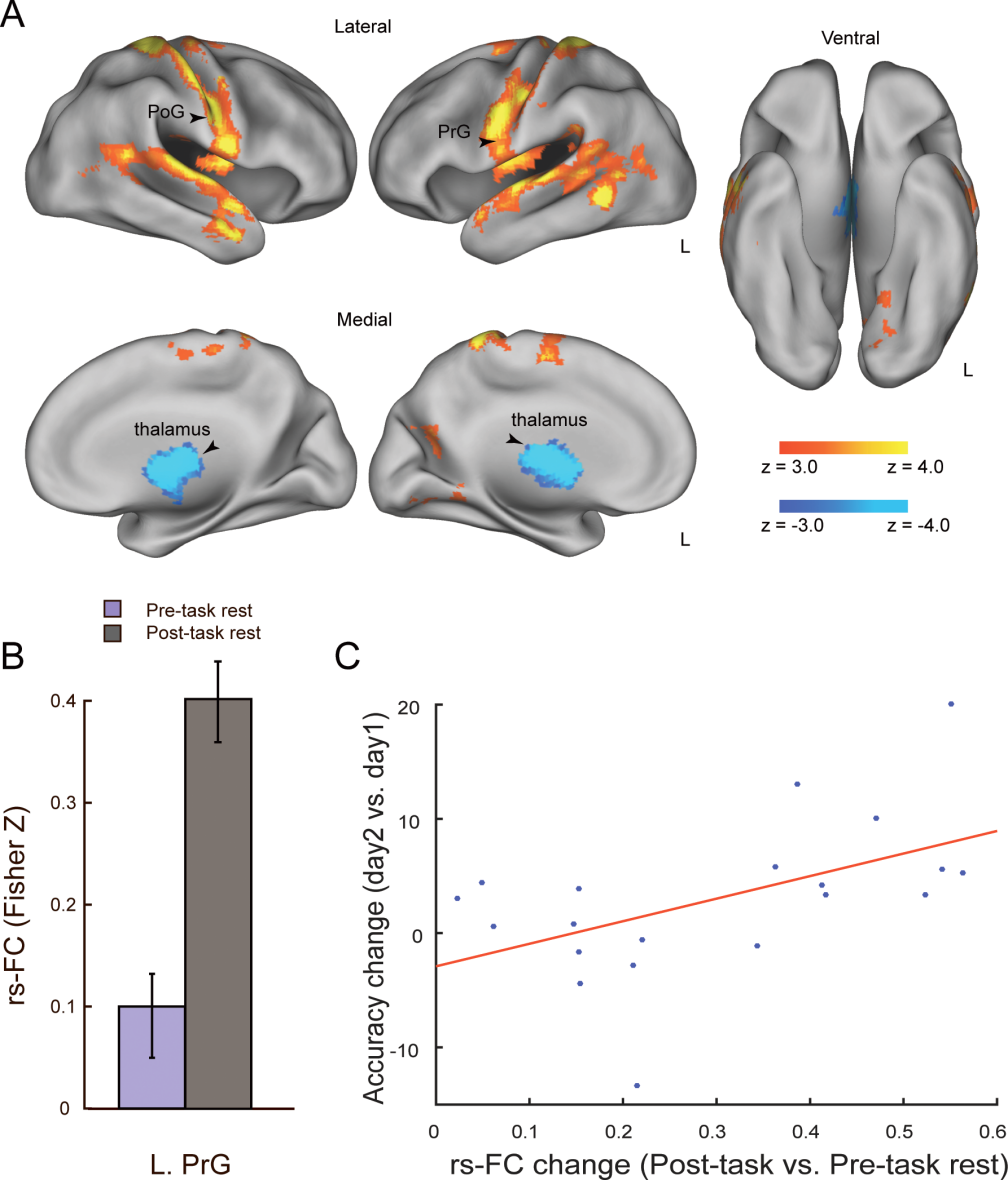
Training-induced rs-FC changes and brain-behavior correlation. (A) The statistical map indicates brain regions showing training-induced rs-FC changes with the MT+ seed (hot: Post-task > Pre-task rest, cold: Pre-task > Post-task rest). The arrow head indicates the left PrG. All maps are thresholded at *P* < 0.05, cluster-level FWE corrected across the whole brain. (B) Pre-task and post-task rs-FC between the MT+ and left PrG. The y axis indicates the correlations (Fisher Z transformed) between the time series extracted from the MT+ and left PrG, averaged across participants. The error bars indicate s.e.m. The bar graph is provided for visualisation purposes, and no statistical test was applied to this data. (C) Scatter plot showing individual differences in behavioural improvement (accuracy gain on day 2 relative to day 1) as a function of training-induced rs-FC change between the MT+ and left PrG. PoG: postcentral gyrus; PrG: precentral gyrus.

To examine training-induced changes in rs-FC, we contrasted seed-based functional connectivity during the pre-task and post-task rs-fMRI sessions. In this analysis, we selected the MT+ as the primary seed that was localized by the parametric effect of the coherence level on the day 1 t-fMRI session (MNI coordinates: *x* = 42 *y* = − 66, *z* = 8). Note that we used only data from day 1 to avoid possible artefacts resulting from differences in head position between day 1 and day 2. First, we obtained rs-FC maps with the MT+ seed separately for the pre-task and post-task rs-fMRI sessions. This revealed that a greater number of voxels located across broad brain regions showed significant rs-FC with the MT+ during the post-task (relative to pre-task) rs-fMRI session (Fig 3). More specifically, we found prominent increases in rs-FC with the MT+ during the post-task (relative to pre-task) rs-fMRI session in the bilateral postcentral gyrus (PoG), the left precentral gyrus (PrG), the left superior temporal gyrus (STG), the left middle temporal gyrus (MTG) and the lateral occipital cortex (LOC) (*P* < 0.05, cluster-level FWE corrected; Fig 4A and Table 2). On the contrary, rs-FC with the MT+ was significantly decreased in subcortical regions (the thalamus and putamen) after the t-fMRI session (Fig 4A and Table 2). Notably, we found no significant change in rs-FC between the pre- and post-task rs-fMRI sessions when we used the dACC, a task-difficulty dependent region, as a seed (*x* = 14, *y* = 22, *z* = 34; *P* > 0.056, cluster-level FWE corrected). Likewise, there were no significant rs-FC changes between the rs-fMRI sessions when we used the right and left insula as seeds (right: *x* = 36, *y* = 22, *z* = 0; *P* > 0.052, cluster-level FWE corrected; left: *x* = − 30, *y* = 30, *z* = 0; *P* > 0.202, cluster-level FWE corrected). As a control analysis, we confirmed that there was no significant rs-FC change when we used the right primary visual cortex, ipsilateral to the MT+ seed, as a control seed ([8 −86 2], 88% BA17, based on meta-analysis database, Neurosynth [http://neurosynth.org/]). These findings suggest that the post-task rs-FC change occurred specifically in the brain regions representing the trained visual feature, but not in the task-difficulty dependent regions or the primary visual cortex. Additionally, we tested whether the training-induced rs-FC changes with the MT+ predicted individuals’ t-fMRI activation changes from day 1 to day 2. Although rs-FC changes were marginally correlated with activation changes in the thalamus and PoG, none of which survived multiple comparison correction (S1–S4 Tables).

**Table 2:** Regions showing significant rs-FC with the MT+ in pre-task rest, post-task rest, and contrasts of post-task > pre-task rest and pre-task > post-task rest (cluster-level FWE-corrected *P* < 0.05). Subpeaks in a given cluster were listed if it survived at *P* < 0.05, voxel-wise FWE corrected across the whole brain.

				MNI coordinates			
Region		Hemi	#voxels	x	y	z	Peak *t*
**Pre-task rest**							
Lateral occipital cortex		R	43483	44	−66	6	23.4
	Precentral gyrus	L		−36	−22	56	10.7
	Cerebelum**	L		−6	−72	−42	7.30
	Postcentral gyrus	R		6	−36	66	6.97
	Supramarginal gyrus	R		60	−36	14	6.77
	Insular cortex	R		40	−2	12	6.67
Supplementary motor cortex		R	1136	6	2	58	6.56
Parietal operculum cortex		L	393	−42	−36	24	5.51
**Post-task rest**							
Lateral occipital cortex		R	70088	44	−66	6	21.1
	Temporal pole	R		28	12	−30	8.69
	Parahippocampal gyrus	L		−30	−6	−24	7.15
	Parahippocampal gyrus	R		20	−2	−20	6.42
	Amygdala*	R		30	−4	−24	6.2
	Precentral gyrus	L		−36	8	20	6.19
	Parahippocampal gyrus	R		22	2	−26	6.16
	Intracalcarine cortex	R		4	−66	12	6
	Thalamus*	R		20	−30	0	5.94
**Post-task rest > Pre-task rest**							
Postcentral gyrus		R	15884	22	−40	66	9.95
	Postcentral gyrus	R		52	−12	50	9.6
	Postcentral gyrus	L		−24	−40	68	8.19
	Inferior temporal gyrus	L		−46	−46	−4	7.62
	Middle temporal gyrus	L		−56	−54	−6	7.49
	Superior temporal gyrus	L		−64	−16	2	6.72
	Planum temporale	L		−52	−18	0	6.59
	Superior frontal gyrus	L		−14	−4	60	6.45
	Postcentral gyrus	R		40	−14	32	6.41
	Middle temporal gyrus	R		52	2	−24	6.37
	Precentral gyrus	L		−54	−4	24	6.32
	Central opercular cortex	R		42	−12	14	6.27
Precuneous cortex		R	217	−10	−68	18	4.68
**Pre-task rest > Post-task rest**							
Thalamus*		L	2163	−6	−2	6	7.79
	Thalamus*	L		−12	−22	14	7.59
	Thalamus*	R		6	−16	12	7.04
	Thalamus*	R		4	−4	6	6.75
	Thalamus*	L		−16	−8	6	6.47
	Thalamus*	R		20	−24	14	6.26
	Putamen*	R		20	16	2	6.25

Anatomical labels derived from Harvard-Oxford cortical structural atlas.

*Labels derived from Harvard-Oxford Subcortical structural atlas.

**Labels derived from Automated Anatomical Labels. L = left hemisphere, R = right hemisphere, #voxels = number of voxels.

### Brain-behavior correlation

Finally, we tested whether the training-induced rs-FC change in the MT+ predicted individuals’ performance improvement (i.e., accuracy gain) on day 2. We found that accuracy gains were positively correlated with training-induced rs-FC changes between the MT+ and left PrG (*r* = 0.522, *P* = 0.018, uncorrected), indicating that ~27% of inter-individual variation in accuracy gains was explained by training-induced rs-FC change between these regions (Fig 4B and 4C). The rs-FC changes in the right PoG also exhibited marginal correlation with accuracy gains (*r* = 0.471, *P* = 0.036, uncorrected). However, given that we observed training-induced rs-FC change with the MT+ in 19 regions, these brain-behavior correlations did not survive multiple comparison correction (see Table 3). We next tested whether the training-induced rs-FC change with the MT+ predicts other aspects of performance improvement (i.e., RT and IE) on day 2 (S5 and S6 Tables). We found that only IE was marginally correlated with training-induced rs-FC changes between the MT+ and the right PoG (*r* = − 0.49, *P* = 0.028, uncorrected), as well as the left PrG (*r* = − 0.480, *P* = 0.034, uncorrected), although these correlations could not survive multiple comparison correction. We further examined whether combinations of two opposing rs-FC changes in cortical and subcortical regions (see Fig 4A) could predict the behavioral learning effects. To do so, we first extracted principal components (PCs) from positive and negative connectivity change maps, then, conducted simple regression analyses to predict the behavioral accuracy using the combined first PCs of each opposing changes. This analysis was also applied to RT and IE. The results revealed no significant regression coefficient for those behavioral indices (accuracy gain: *P* = 0.83, R ^2^ = 0.02; RT: *P* = 0.71, R ^2^ = 0.04; IE: *P* = 0.78, R ^2^ = 0.03). Additionally, we examined whether there were any correlations between behavioral changes within day 1 and rs-FC changes. The analysis revealed no significant correlations.

**Table 3:** Brain-behavior correlation in regions showing training-induced rs-FC changes with MT+ seed: Accuracy gain was used as the behavioral index.

Region	Hemi	*r* value	*P*-value
**Post-task rest > Pre-task rest**			
Postcentral gyrus	R	0.023	0.924
Postcentral gyrus	R	0.089	0.710
Postcentral gyrus	L	0.047	0.844
Inferior temporal gyrus	L	−0.111	0.643
Middle temporal gyrus	L	0.087	0.715
Superior temporal gyrus	L	0.091	0.703
Planum temporale	L	0.133	0.576
Superior frontal gyrus	L	−0.188	0.427
Postcentral gyrus	R	0.471	0.036
Middle temporal gyrus	R	0.063	0.790
Precentral gyrus	L	0.522	0.018
Central opercular cortex	R	0.110	0.645
**Pre-task rest > Post-task rest**			
Thalamus*	L	0.005	0.984
Thalamus*	L	0.145	0.542
Thalamus*	R	0.019	0.935
Thalamus*	R	0.127	0.592
Thalamus*	L	−0.206	0.384
Thalamus*	R	0.263	0.263
Putamen*	R	0.230	0.330

Anatomical labels derived from Harvard-Oxford cortical structural atlas.

*Labels derived from Harvard-Oxford Subcortical structural atlas. L = left hemisphere, R = right hemisphere.

## Discussion

The current study examined whether a brief period of visual perceptual training induced rs-FC changes in feature representation regions immediately after training. Using a visual motion discrimination task, we found that rs-FC with the feature specific area of the MT+ significantly increased in the sensorimotor and temporal cortices and decreased in the subcortical regions after a brief training period (~30 min), indicating a rapid reorganisation of rs-FC in the early learning phase. Importantly, this training-induced offline plasticity was observed specifically in the MT+, but not in higher-order cognitive regions, such as the dACC or insula. Our findings provide new evidence supporting the distinct roles of feature-representation regions and higher-order cognitive regions in VPL [7].

While most previous fMRI studies on VPL have investigated task-related brain activation [2,9], offline processes following task periods are also known to be important for VPL [10,11,22]. For instance, a previous study revealed an fMRI signal increase in the early visual cortex (V1) during sleep after visual perceptual training [12]. This elevated fMRI signal in the feature representation region may indicate spontaneous reactivation of the trained visual feature and the consolidation process during sleep. Recent studies further show that training-induced fMRI signal changes are observed even during wakeful resting periods immediately after the task session [13,14,46]. In particular, Urner *et al*. (2013) [25] showed that brief training on a visual motion discrimination task (~90 min) induced a significant increase in rs-FC between the hippocampus and striatum immediately after training. However, they did not observe training-induced rs-FC changes in visual feature representation regions, leaving it unclear whether visual feature representation regions show offline rs-FC changes during the early learning period. The current study provides the first direct evidence of rapid training-induced rs-FC changes in the MT+ immediately after training on a visual motion discrimination task. One possible explanation for the lack of rs-FC changes in the MT+ in the previous study is that only low-coherence (20%) visual motion stimuli and control static dots were used. In the current study, we used high- as well as low-coherence visual motion stimuli, which might have facilitated offline reactivation in the MT+.

The current finding that training-induced rs-FC changes were specifically observed in the MT+ is of particular interest. One of the long-standing questions regarding the mechanisms underlying VPL is the distinct roles of visual feature-representation regions and higher-order cognitive regions. Some studies have emphasised the critical roles of learning-induced plasticity in visual feature-specific areas (“visual model”), whereas other studies have reported that higher-order cognitive regions involved in decision making (including the dACC) also play key roles (“cognitive model”) [7]. Although both types of region are likely to contribute to VPL [47], recent evidence suggests that specific fMRI signal patterns induced in the early visual cortex during offline periods (i.e., without explicit perceptual discrimination tasks) is sufficient for VPL [48]. Our current findings are consistent with this notion, revealing that the visual feature representation regions are specifically plastic and exhibit rapid rs-FC changes immediately after a brief period of visual perceptual training.

The role of experience-induced rs-FC changes immediately after tasks is a topic of current interest across a range of research domains, including episodic memory encoding and motor learning [13,15,49]. Many recent studies have reported that rs-FC during passive, wakeful rest periods reflect preceding visual/cognitive experience [50–53]. These experience-induced resting-state fMRI signals appear to reflect spontaneous reactivation of recent experiences and offline consolidation processes, thereby contributing to subsequent behavioural performance of memory and learning. For example, a previous study revealed that rs-FC immediately after memory encoding tasks increased in the medial temporal lobe (including the hippocampus), which further predicted memory performance the next day [21]. Another study reported that a short period of sensorimotor learning induced a rapid increase in rs-FC among the fronto-parietal regions and cerebellum [19]. Taken together with the current finding of MT+ specific rs-FC reconfiguration after visual perceptual training, these results suggest that experience-induced plastic changes in rs-FC during a wakeful rest period immediately after task performance may reflect offline processes that are critically important for many different types of memory and learning.

If training-induced rs-FC changes immediately after tasks play a key role in early consolidation processes, rs-FC changes during this period may predict subsequent performance improvements (e.g., ~24 hours after training). Inspired by similar findings in recent memory research [21] we tested this possibility by examining the relationship between training-induced rs-FC changes and performance improvements on day 2 (relative to day 1). We obtained a suggestive result that the rs-FC change between the MT+ and sensory-motor-related regions (i.e., the PrG and PoG) was correlated with behavioural improvement, although this result did not survive multiple comparison correction and should be interpreted with caution. However, this result suggests an interesting hypothesis, that if a perceptual learning task requires fast motor responses, similar to the task used in our study, that task may also induce motor learning, and the resultant learning effects would involve not only relevant sensory areas, but also motor areas. In the current study, we did not have prior knowledge about which brain regions would show performance-dependent rs-FC changes with the MT+. From a post-hoc perspective, our findings seem to suggest that spontaneous coactivation between the MT+ and motor cortex during the post-task rest reflect (or facilitate) the offline consolidation process that associate specific visual features with motor outputs. Our findings may serve as a foundation for future studies to formally test the relationship between training-induced rs-FC changes in specific brain regions during the early learning phase (i.e., immediately after training) and performance improvement in later phases (over days). Moreover, graph analysis [54,55] could be useful for examining the relationship between rapid reorganisation of large-scale functional brain networks immediately after training and subsequent behavioural improvement.

A notable characteristic of the current results is the significant increase in rs-FC between the MT+ and the widespread cortical regions (e.g., sensorimotor and temporal cortices) after training. This is in contrast to the findings of a previous study, which showed a decrease in rs-FC between the visual feature representation regions and dorsal attention system [24]. This apparent discrepancy may be related to the different temporal structure of visual perceptual training and the intervals between training and resting-state fMRI scans. Specifically, the previous study involved several days of intensive visual perceptual training (2–9 days), and examined rs-FC changes well after learning was established. In contrast, the current study focused on the effects of a single session of short-term visual perceptual training (~30 min), and examined rs-FC changes in the early learning phase, immediately after the task session. Previous studies investigating post-task rs-FC change immediately after training have generally reported increased rs-FC in regions specifically related to the task performed [19,25]. Interestingly, previous studies investigating training-dependent changes in task-related fMRI activation have also reported similar distinct profiles depending on early vs. late learning phases [56]. For example, one study showed that V1 activation during the visual perceptual task markedly increased during a relatively early phase of learning, then decreased and returned to baseline during a later learning phase [56]. The current findings may indicate that training-induced rs-FC changes exhibit a similar temporal profile depending on early/late learning phases. This issue is an interesting research target for future studies. It is also notable that we found training-induced decrease in rs-FC between the MT+ and subcortical regions (e.g., the thalamus), which was the opposite from what we observed in the sensorimotor and temporal cortices. According to a previous study, stimulus-induced activation after VPL and one-night sleep was positively correlated with post-training behavioral performance in the PrG and MTG whereas negatively correlated in the thalamus [11]. In line with this previous report, our results suggest opposing contributions of cortical and subcortical regions to offline consolidation processes of VPL.

It has been shown that VPL can be distinguished from visual adaptation, another form of short-term perceptual plasticity, although recent evidence suggests that the boundary between the two types of plasticity is more ambiguous than previously thought [57]. To discriminate VPL from adaptation, one typical experimental procedure is to examine specificity of brain response changes to trained vs. untrained visual features [9,24,56]. However, it is difficult to perform such analyses in the current experimental design. Nonetheless, this issue may be partially addressed by considering that there was no significant activation decrease in area MT+ or behavioral improvement (two characteristic components of adaptation) in the course of the six t-fMRI runs on day 1. These observations suggest that the current results would not involve adaptation effects.

In conclusion, our study revealed that a brief period of visual perceptual training induces rapid rs-FC changes immediately after training in visual feature representation regions, but not in higher-order cognitive regions. This finding provides further support for the distinct roles of visual feature representation regions and decision-related regions in VPL, with a particular emphasis on offline plasticity in feature representation regions during the early learning phase. In a broader context, our study highlights the critical role of experience-induced plasticity during wakeful rest periods immediately after tasks, which may contribute to various types of memory and learning, ranging from VPL, to motor skill acquisition and episodic memory formation [10,22,58].

## Supporting information

S1 Data Data sets for Figs 2 and 4; Table 3; S1–S6 Tables. (XLSX)

S1 Table Task-Rest fMRI correlation in regions showing training-induced rs-FC changes with MT+ seed and MT+ activation change. (DOCX)

S2 Table Task-Rest fMRI correlation in regions showing training-induced rs-FC changes with MT+ seed and dACC activation change. (DOCX)

S3 Table Task-Rest fMRI correlation in regions showing training-induced rs-FC changes with MT+ seed and right insula activation change. (DOCX)

S4 Table Task-Rest fMRI correlation in regions showing training-induced rs-FC changes with MT+ seed and left insula activation change. (DOCX)

S5 Table Brain-behavior correlation in regions showing training-induced rs-FC changes with MT+ seed: Reaction time was used as the behavioral index. (DOCX)

S6 Table Brain-behavior correlation in regions showing training-induced rs-FC changes with MT+ seed: Inverse Efficiency was used as the behavioral index. (DOCX)
