# Supplementary material for: Visual perceptual training reconfigures post-task resting-state functional connectivity with a feature-representation region
Source: PLoS One. 2018 May 9;13(5):e0196866. doi: 10.1371/journal.pone.0196866 (PMC5942817; doi:10.1371/journal.pone.0196866)
Supplement: S1 Table — (DOCX) [file pone.0196866.s002.docx]

**S1 Table**.

| Region | Hemi | *r* value | *P*-value |
| --- | --- | --- | --- |
| **Post- vs. Pre-task rest** |  |  |  |
| Postcentral gyrus | R | −0.278 | 0.235 |
| Postcentral gyrus | R | 0.346 | 0.134 |
| Postcentral gyrus | L | −0.323 | 0.164 |
| Inferior temporal gyrus | L | −0.025 | 0.918 |
| Middle temporal gyrus | L | −0.042 | 0.859 |
| Superior temporal gyrus | L | −0.420 | 0.065 |
| Planum temporale | L | −0.139 | 0.559 |
| Superior frontal gyrus | L | −0.031 | 0.896 |
| Postcentral gyrus | R | 0.171 | 0.471 |
| Middle temporal gyrus | R | −0.107 | 0.653 |
| Precentral gyrus | L | 0.029 | 0.903 |
| Central opercular cortex | R | 0.148 | 0.533 |
| **Post- vs. Pre-task rest** |  |  |  |
| Thalamus^*^ | L | 0.357 | 0.122 |
| Thalamus^*^ | L | 0.472 | 0.035 |
| Thalamus^*^ | R | 0.272 | 0.246 |
| Thalamus^*^ | R | 0.260 | 0.268 |
| Thalamus^*^ | L | 0.562 | 0.010 |
| Thalamus^*^ | R | 0.310 | 0.184 |
| Right Putamen^*^ | R | 0.298 | 0.202 |
