# Supplementary material for: Visual perceptual training reconfigures post-task resting-state functional connectivity with a feature-representation region
Source: PLoS One. 2018 May 9;13(5):e0196866. doi: 10.1371/journal.pone.0196866 (PMC5942817; doi:10.1371/journal.pone.0196866)
Supplement: S2 Table — (DOCX) [file pone.0196866.s003.docx]

**S2 Table**.

| Region | Hemi | *r* value | *P*-value |
| --- | --- | --- | --- |
| **Post- vs. Pre-task rest** |  |  |  |
| Postcentral gyrus | R | −0.504 | 0.023 |
| Postcentral gyrus | R | −0.296 | 0.205 |
| Postcentral gyrus | L | −0.068 | 0.777 |
| Inferior temporal gyrus | L | −0.105 | 0.659 |
| Middle temporal gyrus | L | −0.186 | 0.433 |
| Superior temporal gyrus | L | −0.261 | 0.267 |
| Planum temporale | L | −0.026 | 0.913 |
| Superior frontal gyrus | L | −0.229 | 0.332 |
| Postcentral gyrus | R | −0.196 | 0.407 |
| Middle temporal gyrus | R | 0.007 | 0.978 |
| Precentral gyrus | L | −0.084 | 0.724 |
| Central opercular cortex | R | 0.009 | 0.971 |
| **Post- vs. Pre-task rest** |  |  |  |
| Thalamus^*^ | L | 0.147 | 0.537 |
| Thalamus^*^ | L | 0.049 | 0.837 |
| Thalamus^*^ | R | 0.118 | 0.621 |
| Thalamus^*^ | R | 0.062 | 0.796 |
| Thalamus^*^ | L | 0.046 | 0.846 |
| Thalamus^*^ | R | 0.081 | 0.735 |
| Right Putamen^*^ | R | −0.156 | 0.512 |
