# Supplementary material for: Visual perceptual training reconfigures post-task resting-state functional connectivity with a feature-representation region
Source: PLoS One. 2018 May 9;13(5):e0196866. doi: 10.1371/journal.pone.0196866 (PMC5942817; doi:10.1371/journal.pone.0196866)
Supplement: S3 Table — (DOCX) [file pone.0196866.s004.docx]

**S3 Table**.

| Region | Hemi | *r* value | *P*-value |
| --- | --- | --- | --- |
| **Post- vs. Pre-task rest** |  |  |  |
| Postcentral gyrus | R | −0.457 | 0.043 |
| Postcentral gyrus | R | 0.044 | 0.853 |
| Postcentral gyrus | L | −0.418 | 0.067 |
| Inferior temporal gyrus | L | −0.202 | 0.393 |
| Middle temporal gyrus | L | −0.081 | 0.736 |
| Superior temporal gyrus | L | −0.337 | 0.146 |
| Planum temporale | L | −0.287 | 0.220 |
| Superior frontal gyrus | L | −0.319 | 0.170 |
| Postcentral gyrus | R | −0.029 | 0.903 |
| Middle temporal gyrus | R | −0.231 | 0.328 |
| Precentral gyrus | L | −0.186 | 0.432 |
| Central opercular cortex | R | −0.065 | 0.785 |
| **Post- vs. Pre-task rest** |  |  |  |
| Thalamus^*^ | L | 0.473 | 0.035 |
| Thalamus^*^ | L | 0.316 | 0.174 |
| Thalamus^*^ | R | 0.293 | 0.211 |
| Thalamus^*^ | R | 0.328 | 0.159 |
| Thalamus^*^ | L | 0.397 | 0.083 |
| Thalamus^*^ | R | 0.384 | 0.094 |
| Right Putamen^*^ | R | 0.326 | 0.161 |
