# Supplementary material for: Visual perceptual training reconfigures post-task resting-state functional connectivity with a feature-representation region
Source: PLoS One. 2018 May 9;13(5):e0196866. doi: 10.1371/journal.pone.0196866 (PMC5942817; doi:10.1371/journal.pone.0196866)
Supplement: S4 Table — (DOCX) [file pone.0196866.s005.docx]

**S4 Table**.

| Region | Hemi | *r* value | *P*-value |
| --- | --- | --- | --- |
| **Post- vs. Pre-task rest** |  |  |  |
| Postcentral gyrus | R | −0.531 | 0.016 |
| Postcentral gyrus | R | −0.034 | 0.887 |
| Postcentral gyrus | L | −0.249 | 0.289 |
| Inferior temporal gyrus | L | −0.200 | 0.397 |
| Middle temporal gyrus | L | −0.399 | 0.082 |
| Superior temporal gyrus | L | −0.312 | 0.180 |
| Planum temporale | L | −0.426 | 0.061 |
| Superior frontal gyrus | L | −0.153 | 0.521 |
| Postcentral gyrus | R | 0.028 | 0.908 |
| Middle temporal gyrus | R | −0.170 | 0.474 |
| Precentral gyrus | L | −0.165 | 0.487 |
| Central opercular cortex | R | −0.382 | 0.096 |
| **Post- vs. Pre-task rest** |  |  |  |
| Thalamus^*^ | L | 0.368 | 0.110 |
| Thalamus^*^ | L | 0.096 | 0.686 |
| Thalamus^*^ | R | 0.346 | 0.134 |
| Thalamus^*^ | R | 0.237 | 0.315 |
| Thalamus^*^ | L | 0.223 | 0.344 |
| Thalamus^*^ | R | 0.249 | 0.289 |
| Right Putamen^*^ | R | 0.039 | 0.870 |
