# Supplementary material for: Visual perceptual training reconfigures post-task resting-state functional connectivity with a feature-representation region
Source: PLoS One. 2018 May 9;13(5):e0196866. doi: 10.1371/journal.pone.0196866 (PMC5942817; doi:10.1371/journal.pone.0196866)
Supplement: S5 Table — (DOCX) [file pone.0196866.s006.docx]

**S5 Table**.

| Region | Hemi | *r* value | *P*-value |
| --- | --- | --- | --- |
| **Post- vs. Pre-task rest** |  |  |  |
| Postcentral gyrus | R | 0.243 | 0.301 |
| Postcentral gyrus | R | 0.001 | 0.997 |
| Postcentral gyrus | L | 0.269 | 0.252 |
| Inferior temporal gyrus | L | −0.074 | 0.757 |
| Middle temporal gyrus | L | −0.119 | 0.616 |
| Superior temporal gyrus | L | 0.080 | 0.736 |
| Planum temporale | L | −0.232 | 0.324 |
| Superior frontal gyrus | L | 0.328 | 0.158 |
| Postcentral gyrus | R | −0.168 | 0.479 |
| Middle temporal gyrus | R | −0.090 | 0.706 |
| Precentral gyrus | L | −0.180 | 0.448 |
| Central opercular cortex | R | −0.298 | 0.202 |
| **Post- vs. Pre-task rest** |  |  |  |
| Thalamus^*^ | L | −0.114 | 0.633 |
| Thalamus^*^ | L | −0.310 | 0.184 |
| Thalamus^*^ | R | −0.230 | 0.329 |
| Thalamus^*^ | R | −0.217 | 0.359 |
| Thalamus^*^ | L | −0.061 | 0.797 |
| Thalamus^*^ | R | −0.271 | 0.247 |
| Right Putamen^*^ | R | 0.035 | 0.885 |

Anatomical labels derived from Harvard-Oxford cortical structural atlas (* = Harvard-Oxford subcortical structural atlas). L= left hemisphere, R = right hemisphere.
