# Supplementary material for: Visual perceptual training reconfigures post-task resting-state functional connectivity with a feature-representation region
Source: PLoS One. 2018 May 9;13(5):e0196866. doi: 10.1371/journal.pone.0196866 (PMC5942817; doi:10.1371/journal.pone.0196866)
Supplement: S6 Table — (DOCX) [file pone.0196866.s007.docx]

**S6 Table**.

| Region | Hemi | *r* value | *P*-value |
| --- | --- | --- | --- |
| **Post- vs. Pre-task rest** |  |  |  |
| Postcentral gyrus | R | 0.017 | 0.943 |
| Postcentral gyrus | R | −0.116 | 0.626 |
| Postcentral gyrus | L | 0.066 | 0.783 |
| Inferior temporal gyrus | L | 0.095 | 0.690 |
| Middle temporal gyrus | L | −0.110 | 0.645 |
| Superior temporal gyrus | L | − 0.046 | 0.848 |
| Planum temporale | L | −0.198 | 0.403 |
| Superior frontal gyrus | L | 0.218 | 0.356 |
| Postcentral gyrus | R | −0.490 | 0.028 |
| Middle temporal gyrus | R | −0.065 | 0.785 |
| Precentral gyrus | L | −0.480 | 0.032 |
| Central opercular cortex | R | −0.223 | 0.344 |
| **Post- vs. Pre-task rest** |  |  |  |
| Thalamus^*^ | L | −0.072 | 0.763 |
| Thalamus^*^ | L | −0.260 | 0.269 |
| Thalamus^*^ | R | −0.120 | 0.615 |
| Thalamus^*^ | R | − 0.212 | 0.369 |
| Thalamus^*^ | L | 0.071 | 0.765 |
| Thalamus^*^ | R | −0.317 | 0.174 |
| Right Putamen^*^ | R | −0.220 | 0.352 |
